# Supplementary material for: Genetic structure of black soldier flies in northern Iran
Source: PLoS One. 2024 Aug 15;19(8):e0308953. doi: 10.1371/journal.pone.0308953 (PMC11326627; doi:10.1371/journal.pone.0308953)
Supplement: Attachment — Submitted filename: PONE-D-24-11332.pdf [file pone.0308953.s001.pdf]

PLOS ONE

Mitochondrial and nuclear DNA revealed first record of Hermetia illucens (Diptera: Stratiomyidae) in Northern Iran

--Manuscript Draft--

**Manuscript Number:**	PONE-D-24-11332
**Article Type:**	Research Article
**Full Title:**	Mitochondrial and nuclear DNA revealed first record of Hermetia illucens (Diptera: Stratiomyidae) in Northern Iran
**Short Title:**	genetic structure of Black solder in Northern Iran
**Corresponding Author:**	Mohammad Ali Oshaghi<br>Tehran University of Medical Sciences<br>Tehran, Tehran IRAN, ISLAMIC REPUBLIC OF
**Keywords:**	Black soldier fly; COI; rDNA; decomposer; organic waste; dietary source; Iran
**Abstract:**	Background: The black soldier fly, Hermetia illucens, is known for nutrient-recycling through the bioconversion of organic waste into protein-rich insect.<br>Methods and Results: We directed a survey on the Caspian Sea coast, with a reconstructing demographic relationships study using two parts of mitochondrial cytochrome C oxidase 1 (COI) gene (barcode and 3' end regions) and nuclear internal transcribed spacer 2 (ITS2) to identify H. illucens' genetic diversity in retrospect to the global diversity and the potential origin of the Iranian BSF population. Larvae and adults were recovered from highly decomposed poultry manure, in May 2020. Sequence analysis of both regions of COI gene (about 1500 bp) revealed a single haplotype, identical to that of haplotype C, a worldwide commercial strain originated from Nearctic, Palearctic, or African biogeographic regions. However, the ITS2 locus was confirmed to be invariable across samples from diverse biogeographic regions.<br>Conclusion: The results proved the presence of H. illucens in north of Iran, however, dispersal of the black soldier fly may not be restricted to the coast of Caspian Sea due to heavy international trading in southern coasts of the country. The introduction and settlement of this fly in other parts of the country is highly suggested.
**Order of Authors:**	Afrooz Boukan, MSc
	Jamasb Nozari, PhD
	Fahimeh Talebzadeh
	Nazanin Neseri Karimi
	Katayoun Pahlavan Yali, MSc
	Mohammad Ali Oshaghi
**Additional Information:**	
**Question**	**Response**
**Financial Disclosure**<br><br>Enter a financial disclosure statement that describes the sources of funding for the work included in this submission. Review the submission guidelines for detailed requirements. View published research articles from *PLOS ONE* for specific examples.<br><br>This statement is required for submission	Yes

and **will appear in the published article** if the submission is accepted. Please make sure it is accurate.<br><br>**Funded studies**<br>Enter a statement with the following details:<br>• Initials of the authors who received each award<br>• Grant numbers awarded to each author<br>• The full name of each funder<br>• URL of each funder website<br>• Did the sponsors or funders play any role in the study design, data collection and analysis, decision to publish, or preparation of the manuscript?<br><br>Did you receive funding for this work?	
Please add funding details.<br>as follow-up to "**Financial Disclosure**<br><br>Enter a financial disclosure statement that describes the sources of funding for the work included in this submission. Review the submission guidelines for detailed requirements. View published research articles from *PLOS ONE* for specific examples.<br><br>This statement is required for submission and **will appear in the published article** if the submission is accepted. Please make sure it is accurate.<br><br>**Funded studies**<br>Enter a statement with the following details:<br>• Initials of the authors who received each award<br>• Grant numbers awarded to each author<br>• The full name of each funder<br>• URL of each funder website<br>• Did the sponsors or funders play any role in the study design, data collection and analysis, decision to publish, or preparation of the manuscript?<br><br>Did you receive funding for this work?"	Tehran University of Medical Sciences

Please select the country of your main research funder (please select carefully as in some cases this is used in fee calculation).<br>as follow-up to "**Financial Disclosure**<br><br>Enter a financial disclosure statement that describes the sources of funding for the work included in this submission. Review the submission guidelines for detailed requirements. View published research articles from *PLOS ONE* for specific examples.<br><br>This statement is required for submission and **will appear in the published article** if the submission is accepted. Please make sure it is accurate.<br><br>**Funded studies**<br>Enter a statement with the following details:<br>• Initials of the authors who received each award<br>• Grant numbers awarded to each author<br>• The full name of each funder<br>• URL of each funder website<br>• Did the sponsors or funders play any role in the study design, data collection and analysis, decision to publish, or preparation of the manuscript?<br><br>Did you receive funding for this work?"	IRAN, ISLAMIC REPUBLIC OF - IR
**Competing Interests**<br><br>Use the instructions below to enter a competing interest statement for this submission. On behalf of all authors, disclose any competing interests that could be perceived to bias this work—acknowledging all financial support and any other relevant financial or non-financial competing interests.<br><br>This statement is required for submission and **will appear in the published article** if the submission is accepted. Please make sure it is accurate and that any funding	The authors have declared that no competing interests exist.

sources listed in your Funding Information later in the submission form are also declared in your Financial Disclosure statement.<br><br>View published research articles from *PLOS ONE* for specific examples.<br><br>**NO authors have competing interests**<br><br>Enter: *The authors have declared that no competing interests exist.*<br><br>**Authors with competing interests**<br><br>Enter competing interest details beginning with this statement:<br><br>*I have read the journal's policy and the authors of this manuscript have the following competing interests: [insert competing interests here]*<br><br>* typeset	
**Ethics Statement**<br><br>Enter an ethics statement for this submission. This statement is required if the study involved:<br><br>• Human participants<br>• Human specimens or tissue<br>• Vertebrate animals or cephalopods<br>• Vertebrate embryos or tissues<br>• Field research<br><br>Write "N/A" if the submission does not require an ethics statement.<br><br>General guidance is provided below. Consult the submission guidelines for detailed instructions. **Make sure that all information entered here is included in the Methods section of the manuscript.**	This article does not contain any studies with human participants or animals performed by any of the authors. The protocols were conducted in this study followed the guidelines of the institutional ethical committee (University of Tehran).

<table>
<tr>
<td>
Format for specific study types

Human Subject Research (involving human participants and/or tissue)
• Give the name of the institutional review board or ethics committee that approved the study
• Include the approval number and/or a statement indicating approval of this research
• Indicate the form of consent obtained (written/oral) or the reason that consent was not obtained (e.g. the data were analyzed anonymously)

Animal Research (involving vertebrate animals, embryos or tissues)
• Provide the name of the Institutional Animal Care and Use Committee (IACUC) or other relevant ethics board that reviewed the study protocol, and indicate whether they approved this research or granted a formal waiver of ethical approval
• Include an approval number if one was obtained
• If the study involved non-human primates, add additional details about animal welfare and steps taken to ameliorate suffering
• If anesthesia, euthanasia, or any kind of animal sacrifice is part of the study, include briefly which substances and/or methods were applied

Field Research

Include the following details if this study involves the collection of plant, animal, or other materials from a natural setting:
• Field permit number
• Name of the institution or relevant body that granted permission
</td>
<td></td>
</tr>
<tr>
<td>
Data Availability

Authors are required to make all data underlying the findings described fully available, without restriction, and from the time of publication. PLOS allows rare exceptions to address legal and ethical concerns. See the PLOS Data Policy and FAQ for detailed information.
</td>
<td>Yes - all data are fully available without restriction</td>
</tr>
</table>

A Data Availability Statement describing where the data can be found is required at submission. Your answers to this question constitute the Data Availability Statement and **will be published in the article**, if accepted.<br><br>**Important:** Stating 'data available on request from the author' is not sufficient. If your data are only available upon request, select 'No' for the first question and explain your exceptional situation in the text box.<br><br>Do the authors confirm that all data underlying the findings described in their manuscript are fully available without restriction?	
**Describe where the data may be found in full sentences. If you are copying our sample text, replace any instances of XXX with the appropriate details.**<br><br>• If the data are **held or will be held in a public repository**, include URLs, accession numbers or DOIs. If this information will only be available after acceptance, indicate this by ticking the box below. For example: *All XXX files are available from the XXX database (accession number(s) XXX, XXX.).*<br>• If the data are all contained **within the manuscript and/or Supporting Information files**, enter the following: *All relevant data are within the manuscript and its Supporting Information files.*<br>• If neither of these applies but you are able to provide **details of access elsewhere**, with or without limitations, please do so. For example:<br><br>*Data cannot be shared publicly because of [XXX]. Data are available from the XXX Institutional Data Access / Ethics Committee (contact via XXX) for researchers who meet the criteria for access to confidential data.*<br><br>*The data underlying the results presented in the study are available from (include the name of the third party*	The sequence data in this manuscript are available in GenBank database with the following accession numbers OQ803472-<br>OQ803477

and contact information or URL).<br>• This text is appropriate if the data are owned by a third party and authors do not have permission to share the data.<br><br>* typeset	
Additional data availability information:	

manuscript Click here to access/download;Manuscript;manuscript1.docx

Mitochondrial and nuclear DNA revealed first record of *Hermetia illucens* (Diptera: Stratiomyidae) in Northern Iran

Afrooz Boukan[1], Jamasb Nozari[*1], Nazanin Naseri Karimi[2], Fahimeh Talebzadeh[2], Katayoun Pahlavan Yali[1], Mohammad Ali Oshaghi[2*]

[1]*Department of Plant Protection, Faculty of Agriculture and Natural Resources, University of Tehran, Karaj, Iran,*

[2]*Departement of Medical Entomology and Vector Control, School of Public Health, Tehran University of Medical Sciences, Tehran, Iran,*

Email ;

afrooz.boukan@ut.ac.ir,

nozari@ut.ac.ir;

n-naseri@razi.tums.ac.ir

f_talebzadeh@razi.tums.ac.ir

Kpahlavanyali@yahoo.com

moshaghi@tums.ac.ir

[C]orresponding authors: M.A. Oshaghi

E-mail: moshaghi@sina.tums.ac.ir

OrcID: http://orcid.org/0000-0003-3004-0364

[*] Co-corresponding authors: E-mail: moshaghi@sina.tums.ac.ir

Mitochondrial and nuclear DNA revealed first record of *Hermetia illucens* (Diptera: Stratiomyidae) in Northern Iran

Abstract

Background: The black soldier fly, *Hermetia illucens*, ~~is known for nutrient-recycling through the bioconversion of organic waste into protein-rich insect.~~

Methods and Results: We directed a survey on the Caspian Sea coast, with a reconstructing demographic relationships study using two parts of mitochondrial cytochrome C oxidase 1 (COI) gene (barcode and 3' end regions) and nuclear internal transcribed spacer 2 (ITS2) to identify *H. illucens'* genetic diversity in retrospect to the global diversity and the potential origin of the Iranian BSF population. Larvae and adults were recovered from highly decomposed poultry manure, in May 2020. Sequence analysis of both regions of COI gene (about 1500 bp) revealed a single haplotype, identical to that of haplotype C, a worldwide commercial strain originated from Nearctic, Palearctic, or African biogeographic regions. However, the ITS2 locus was confirmed to be invariable across samples from diverse biogeographic regions.

Conclusion: The results proved the presence of *H. illucens* in north of Iran, however, dispersal of the black soldier fly may not be restricted to the coast of Caspian Sea due to heavy international trading in southern coasts of the country. The introduction and settlement of this fly in other parts of the country is highly suggested.

Keywords: Black soldier fly; COI; rDNA; decomposer; organic waste; dietary source; Iran

Introduction

Hermetia illucens (L.) (Diptera, Stratiomyidae) is known as the black soldier fly (BSF) and is a promising candidate for nutrient-recycling through the bioconversion of organic waste into

biomass and the insect farming industry [1-2]. BSF is known as a dietary source of insect protein worldwide [3-4]. Farming BSF on various organic wastes has been suggested as an excellent opportunity for producing nutrient-rich animal organic fertilizer, feed, fuel, and bio-based products [5,6]. At present there are several registered trademarks of the larvae, that are used as food in poultry and aquaculture farms [7-10, also see https://phoenixworm.com]. The larvae of this species are active decomposers of various rotting organic matter, including fruit and vegetable waste, agro-industrial by-products, compost, organic leachates, corpses, and food waste [11-14]. Some larval by-products of this fly are also used for eradicating multidrug-resistant bacteria in aquaculture [15]. BSF are considered to be a possible source of antimicrobial peptides (AMPs) for substitution of antibiotics in livestock farming, which is beneficial to human and animal welfare [16-17]. BSF, as a sarcosaprophagous insect, has been used for estimation of postmortem interval (PMI) in forensic investigations [18]. From a public health perspective, BSF larvae are thought to prevent infestations by house flies, *Musca domestica* L. (Diptera: Muscidae), seeking oviposition sites that lead to reduction of the house fly population [19]. The larvae have six larval stages before pupation and adult females lay 500–1000 eggs [20].

Although this species is cosmopolitan and reported in many different biogeographical regions of the world [21], it is believed that the species originates in the New World [2, 22]. Therefore, the Old-World populations are considered non-indigenous, and thought to be mainly introduced by shipping [22]. In Asia, this species was first reported from Malaysia in the 1940s and later from China in 1960 [22]. However, information on species distribution and abundance in Iran is scarce: the species appears to have been introduced to the country through international trade in the region over the last decades. It is known that many of the initially known BSF habitats outside the New World are near to the coast or on islands [23].

Studying of cytochrome oxidase subunit, I gene of mitochondrial DNA (mtDNA-COI) has been used as an advanced systematic tool applied in evolutionary and population study on species taxonomy and delimitation [24]. This gene is one of the biggest genes in the metazoan mitochondrial genome and it is characterized by higher variability than the other mitochondria genes. It is also one of the most important molecular markers used for molecular taxonomy and systematic of living things and microorganisms [25-28]. Of different segments of the COI gene, DNA barcode region at 5’ end as well as the 3’ end of the gene are used vastly in molecular systematic in literature [29-34]. The barcoding region provides a common source of DNA sequence for taxonomy and identification of organisms [35], whereby scientists can compare living organisms. ~~Also~~, the internal transcribed spacer II (ITS2) ribosomal DNA locus has previously been applied to enhance species identification of different insects including beetles [36], ticks [37], sand flies [38], and mosquitoes [26,39-40].

Here we carried out the first survey, across the Caspian Sea coast in northern Iran, to find the BSF species. We investigated the morphological characteristics of the field-collected flies during the summer 2020, and then determined their genetic structure, using two mitochondrial and a nuclear molecular marker: the 5’ end of cytochrome oxidase I (COI), known as the barcode region, the 3’ end of COI, and the ITS2.

Material and methods

Study area, sample collection, and species identification

The study was conducted along the Caspian Sea coast in Mazandaran and Gilan, two Northern provinces of Iran. ~~Adult and larval black soldier flies~~ were captured respectively with an insect net and forceps ~~manually~~ in autumn (temperature 10°C and relative humidity (RH) 65%) and winter (temperature 3°C and RH 60%), from a residential area in the Caspian region, and near decaying

organic matter, particularly poultry manure and slaughterhouse waste. Some specimens were kept alive for cultivation, and a subset of specimens was stored in 70% ethanol at 4°C until analysis. The collected specimens were transferred to the Bio-systematic Entomology Laboratory of the University of Tehran for species identification. Morphological identification of specimens was performed using the key of de Carvalho and De Mello-Patiu [41]. To dissect the genitals, the two extremities of the abdomen were removed with a dissection needle and cleaned with 10% potassium hydroxide (KOH). Slides were then prepared from the genitals with Mount Canada balsam solution. The main characteristics of this species included two segmented cerci, wing pattern, naked eyes, and the pattern on the eyes (Figure 1). Illustrations were made using a ZEISS Standard Microscope and Optika SZM 1 stereomicroscope equipped with a Sony TM digital camera. Finally, the species diagnosis was confirmed by senior insect biosystematist, Dr. Martin Hauser from California Department of Food and Agriculture, Plant Pest Diagnostics Branch, USA. A subset of morphologically identified specimens was selected for molecular analysis in the Insect Molecular Biology Lab, Department of Biology of Vectors and Control of Diseases, Tehran University of Medical Sciences.

Molecular analysis

Prior to DNA extraction, adult black solder flies were individually rinsed twice with 70% ethanol, centrifuged at 12,000 RPM for 2 min and left to dry in the air. One thorax of an individual fly was used for DNA extraction. The dried thorax was ground with sterile pestles in DNA lysis buffer. DNA extraction was performed using a commercial kit (Qiagen DNeasy Blood and Tissue kit, Hilden, Germany), according to the manufacturer's instructions. From this specimen DNA, two parts of the mitochondrial protein-coding COI gene and the nuclear ITS2 region were amplified. The first amplicon was an 884-bp region of the 3' end of the COI gene, which was amplified by

polymerase chain reaction (PCR), using the primers C1-J-2183: (5′-CAACATTTATTTTGATTTTTTGG-3′) and T1-2-n-3014 (5′-CCATTGCATCTGCCATATTA-3′) (Simon et al. 1994). The second amplicon was a 709-bp region of 5’ end of the COI gene, known as the COI barcode region [25], which was amplified using the universally conserved primer pairs LCO-1490 (5’-GGTCAACAAATCATAAAGATATTGG-3’) and HCO-2198 (5’TAAACTTCAGGGTGACC AAAAAATCA-3’) [42]. The third amplicon was the ITS2 rDNA locus which was amplified and sequenced using the thermal cycling program and primers (5.8S: 5’-TGTGAACTGCAGGACACAT-3’ and 28S: 5’-TATGCTTAAATCAGGGGGT-3’) reported by Ståhls et al [21].

All PCR reactions were conducted in a total volume of 25μL, using the Taq DNA Polymerase 2x Master Mix RED, Ampliqon (Denmark), with the following reagents: 1–2μL of DNA extract, 12.5μL of Master mix, 1μL of each primer (10mM), and 8.5–9.5μL of sterile water. For both COI regions, the PCR thermal profile was 2 min at 94°C, followed by 5 cycles of 94°C (30s), 45°C (40s), and 72°C (60s), and then 35 cycles of 94°C (30s), 51°C (40s), and 72°C (60s), with a final extension at 72°C for 10 min. PCR products were separated by 1% agarose gel electrophoresis, followed by Green Viewer staining and imaging using a UV transilluminator. Successful amplicons were sequenced bidirectionally by the Genetic Codon Company, Tehran, Iran, using the same primers as for amplification.

Analysis of DNA sequences

The quality of crude sequences was improved using the Chromas 2.6.5 program by removing areas of poor quality at each end of the sequences. The consensus of confident sequences was then examined using the NCBI (nucleotide collection) database. Multiple sequences were aligned using the Clustal Omega package [43]. For phylogenetic analysis, a representative barcode or 3’ end

sequence of the Iranian black soldier fly were compared with those barcode (n=36) or 3' end (n=17) publically-available DNA from Genbank databases, which represent different haplotypes [44] and/or world biogeographic regions, including Nearctic, Neotropical, Palearctic, East-Palearctic, Afrotropical, Oriental, and Australasia, as already described by Ståhls et al. [21] and Guilliet et al. [44] (Table 1 and Table 2). Phylogenetic analysis was performed using the Neighbor-Joining method embedded in MEGA X [45].

Table 1. The sequence data of 5′ ends of the COI barcode region was used for phylogenetic analysis in this study. DS: direct submission.

Biogeographical Region	Haplotype*	Continent	Country	GenBank ID number	Reference
Palearctic	C	Asia	Iran	OQ803472	This study
ORIENTAL	C	ASIA	Bhutan	LR778160	[21]
ORIENTAL	E	ASIA	Singapore	MT178485	[21]
ORIENTAL	E	ASIA	Bhutan	LR778159	[21]
ORIENTAL	U	ASIA	Thailand	LR778162	[21]
ORIENTAL	A	ASIA	Malaysia	LR792223	[21]
AFROTROPICAL	C	AFRICA	Kenya	LR778157	[21]
AFROTROPICAL	G	AFRICA	Benin	MT151287	[21]
AFROTROPICAL	A	AFRICA	La Reunion	MT151288	[21]
AFROTROPICAL	U	AFRICA	South Africa	MT178507	[21]
AFROTROPICAL	G	AFRICA	Ghana	LR778158	[44]
PALEARCTIC	C	EUROPE	Spain	LR792260	[21]
PALEARCTIC	A	EUROPE	Switzerland	LR778207	[21]
PALEARCTIC	F	EUROPE	Switzerland	LR792261	[21]
PALEARCTIC	U/C	EUROPE	France	MT178493	[21]
PALEARCTIC	C	EUROPE	Netherlands	MT483933	DS
PALEARCTIC	C	EUROPE	Russia	KY817115	DS
EAST_PALEARCTIC	C	ASIA	China	NC_035232	DS
EAST_PALEARCTIC	F	ASIA	S. Korea	FJ794401	[44]
EAST_PALEARCTIC	B	ASIA	S. Korea	HQ541188	[47]
EAST_PALEARCTIC	A	ASIA	S. Korea	HQ541228	[47]
NEOTROPICAL	D	America	Mexico	LR778208	[21]
NEOTROPICAL	H	America	Bolivia	LR778193	[44]
NEOTROPICAL	A	America	Bolivia	LR792195	[21]
NEOTROPICAL	U	America	Colombia	LR778204	[21]
NEOTROPICAL	U	America	Peru	LR778213	[21]
NEOTROPICAL	U	America	Peru	LR792231	[44]

NEOTROPICAL	U	America	Brazil	LR778206	[21]
NEOTROPICAL	H	America	Brazil	LR792236	[44]
NEARCTIC	U/C	America	USA	MT178490	[21]
NEARCTIC	U	America	USA	MT178458	[21]
NEARCTIC	E	America	Canada	KM967419	[48]
AUSTRALASIA	U	AUSTRALIA	AUSTRALIA	LR792227	[21]
AUSTRALASIA	E	AUSTRALIA	AUSTRALIA	MT178508	[21]
AUSTRALASIA	A	AUSTRALIA	AUSTRALIA	LR792225	[21]
AUSTRALASIA	E	AUSTRALIA	AUSTRALIA	LR792256	[21]

*: Based on ten major COI haplotypes (A-J, and U: unknown) suggested by Guilliet et al. [44]. /: presumably.

Table 2. The sequence data of 3′ ends of the COI gene (840 bp) was used for phylogenetic analysis in this study.

Biogeographical Region	Haplotype *	Continent	Country	GenBank ID Number/code**	Reference
PALEARCTIC	C	Asia	Iran	OQ803473-OQ803477	This study
AFROTROPICAL	C	AFRICA	Kenya	a7	[44]
AFROTROPICAL	C	AFRICA	Kenya	a25	[44]
AFROTROPICAL	C	AFRICA	Ghana	a2	[44]
PALEARCTIC	A	EUROPE	France	a11	[44]
PALEARCTIC	H	EUROPE	France	a39	[44]
PALEARCTIC	J	EUROPE	France	a48	[44]
PALEARCTIC	F	EUROPE	France	a52	[44]
PALEARCTIC	C	EUROPE	France	a12	[44]
PALEARCTIC	C	EUROPE	France	a10	[44]
PALEARCTIC	H	EUROPE	Guyane	a16	[44]
PALEARCTIC	C	EUROPE	UK	a12	[44]
EAST_PALEARCTIC	C	ASIA	Taiwan	a8	[44]
EAST_PALEARCTIC	C	ASIA	China	NC_035232	[47]
NEOTROPICAL	D	America	Mexico	a15	[44]
NEARCTIC	C	America	USA	Berkly_female	[44]
	Outgroup			*Exaireta spinigera*	[44]

*: Based on seven major mitochondrial genome haplotypes (A-C-D-F-G-H-J) suggested by Guilliet et al 2022. **: Adapted from Guilliet et al. [44].

Results

Our morphological investigation revealed the presence of a field population of *H. illucens* in the Caspian coast region in northern Iran. A subset of sampled specimens from that population was randomly selected for molecular analysis, and the remaining specimens (larvae or adults) were kept alive and used for population rearing.

COI 3'-end and barcode analyses

The PCR amplicons were produced successfully for both 5'- and 3'- parts of the COI gene. The COI dataset sequences for both regions were obtained for a subset (n=5) of the field-collected specimens. After trimming, the sequences of about 628 bp in length for the 5' end and 840 bp in length for the 3'-end of the gene, respectively, were obtained. The A + T contents were 62.0 and 65.95% (mean 63.97%) respectively for 5'-end (barcode) and 3'-end regions. All sequences from this population were identical.

The sequence data were compared with a number of publically-available COI DNA barcodes and 5'-end regions from Genbank sequence databases. This analysis showed that the barcode region of Iranian BSF specimens were identical to their counterparts in China, USA, Bhutan, Russia, Spain, France, and Netherlands from various biogeographical regions comprising East-Palearctic, Nearctic, Oriental, and Palearctic.

When the sequences of the 3'-end of the COI gene were compared with available data in GenBank or literature [44], the Iranian BSF population matched with various samples from USA, China, Taiwan, UK, France, Kenya, and Ghana. Further analysis revealed that the Iranian specimens were 100% identical to haplotype C (Table 2), which corresponds to globally common commercial strains [21, 44].

Phylogenetic analysis

This analysis showed that the maximum sequence divergence among the haplotypes was 4.3% for 5’ end (628 bp) and 5.83% for 3’ end (840 bp) of COI gene. The Neighbor-Joining phylogenetic analysis for the COI barcode region could resolve the specimens into two main clades (Fig. 1), each containing two subclades. The Iranian specimen (Palearctic region) was sister taxon with representative specimens from East-Palearctic, Palearctic, Oriental, and Nearctic regions. The Iranian specimen was associated with the commercial specimens (haplotype C) from China, Netherlands, Russia, Bhutan, Spain, France, and USA. Haplotype B was the closest haplotype to haplotype C. Other haplotypes including A, D, E, G, and H were positioned in a separate clade comprising representatives from Neotropical, Nearctic, Afrotropical, Australasia, and Oriental regions while haplotype F samples were grouped in both main clades (Fig. 2).

The phylogenetic analysis for the 3’ end of COI gene showed similar topography with the tree inferred from the 5’ end of the COI gene (Fig. 2). Similarly, the Iranian BSF population was associated with the commercial specimens (haplotype C) from USA, China, Taiwan, UK, France, Kenya, and Ghana and altogether constituted a distinct clade far from other haplotypes. Other haplotypes including A, D, G, H, and J were positioned in a separate clade comprising representatives from Neotropical, Nearctic, Afrotropical, Australasia, and Oriental regions (Fig. 3).

ITS2 rDNA analyses

The PCR amplicons (about 453 bp) were produced successfully for ITS2 region. The ITS2 sequences were obtained for a subset (n=5) of the field-collected specimens. After trimming, the sequences of about 433 bp in length were obtained. The sequences of all five specimens were

identical. The sequence data were compared with all publicly-available ITS2 DNA regions from GenBank sequence databases. This analysis showed that the ITS2 region of Iranian BSF specimens were identical to their counterparts from various biogeographical regions comprising Neotropical, Nearctic, Afrotropical, and Palearctic. Because the ITS2 rDNA sequences displayed no intraspecific variation among sequences, no phylogenetic analysis was performed for the ITS2 sequences.

Discussion

Here in this study, we report the presence of the black soldier fly (BSF), *H. illucens,* in northern Iran based on morphological characteristics and molecular analyses of COI and ITS2 loci. This finding is important for people who are involved in agriculture, aquaculture, animal husbandry, and poultry-keeping, as well as public health and waste management. In northern Iran the available lands and water are limited and, furthermore, waste management has become a great public health problem for more than a decade [49]. Finding the BSF in north of Iran is promising for people involved in organic waste recycling.

Animal farming, particularly poultry, is one of the main activities of people in northern Iran. Investment in the rearing of BSF and establishing a cultured strain from this field population seems convenient in in these areas, since black soldier fly larvae (BSFL) are a valuable biomass and can provide protein-rich feed for animals. The black soldier fly larvae are known to be greedy feeders that ingest and degrade most organic materials, from 55 up to 70% [50-51]. The larvae can consume everything, from agriculture by-products [16, 52] and manure [20, 53-54] to animal remains [55-56]. These wastes can then be converted into high-value insect biomass that is rich in both proteins and fats and can be used as feed for the poultry and aquaculture industries [57-58]. The insect biomass can also reduce the requirement for certain feed nutrients (e.g., soybean) in

animal farming [58]. Also, current studies have revealed the insect's ability to tolerate microplastics (MP) and consider the possibility that they may be able to degrade polymers [59].

In this study we performed an initial survey for the presence of BSF on the Caspian Sea coast in northern Iran. This fly has great ecological and economic value, and the economic value and the ecological consequences of mass-producing this fly in an Iranian context would be worth investigating. Since no reports of Stratiomyidae were made in the Middle East so far [60] the present study, which is based on morphological and molecular diagnosis on the population of *H. illucens* caught from nature, is the first report of the entry and establishment of this species in the Middle East ecosystem. It remains unclear whether the investigated population can be considered naturalized in Northern Iran or is subject to recurrent yet temporary occurrences, possibly going back to flies escaping from BSF rearing facilities in the wider region. Next to conservative mitochondrial genes and low/lack intraspecific variation of ITS2 region, higher resolving nuclear genetic markers, such whole genome sequencing or microsatellites [2], could elucidate its status as being established versus occasionally present in the region, and contribute to our understanding on genotype-by-environment interactions underlying ecological adaptation and associated phenotypic variation [62]. Ecosystem sustainability is based on the balance and natural relationships of organisms and their physical environment (ecological adaptation), and the boundaries of species present are determined based on geographical and ecological constraints, the needs of each species, the ability to spread and compete with other species. Some species enter an ecosystem other than the natural distribution range beyond their normal distribution range due to human activities (intended or unintended) and show different behaviors in the new conditions and may be able to adapt in this new habitat. Many of these species do not interfere with the new habitat and are not known as invasive species but find a niche like indigenous species. Introduced

species become invasive species when they compete with indigenous species for available resources and outcompete native species from their original habitat. Humans have disrupted geographical pattern of such species by moving them around the world [63]. The probability of success of an introduced species in a new region is about one out of ten. Of course, one out of ten imported species can survive in nature and one out of ten established species can become a pest [28, 64]. Invasive introduced species are a major threat to biodiversity today [65]; however, since *H. illucens* is an introduced species that may lack competition with native species and can have a positive effect on cleaning the environment from toxins, organic waste, etc., it provides a wide range of usability with environmental approaches in the region while rejecting the possibility of invasion. However, it is not possible to determine with certainty when and where this species first established in Iran, and they have likely been transferred to nature due to the existence of companies importing and breeding such flies in some provinces such as Mazandaran and Khorasan. However, not finding this species in the mentioned provinces deserves further questions and studies. One reason for not finding them in the direct vicinity of these farms may be resource availability or even adaptation of farmed BSF to specific resources not available close by. Further studies including complete genome sequencing and polymorphic nuclear genetic markers such as microsatellites could provide more information compared to the available databases [2]. Based on the present observations, this insect, originally preferring tropical climates, may exhibit cyclic occurrence in northern Iran, with a preference for cooler yet comparatively humid autumn, reiterating its immense adaptive potential to a wide range of environmental conditions. It is worth noting that the higher temperature within compost heaps may protect larvae from freezing to death, and allow them to survive through a diapause-like phase.

We found the mitochondrial haplotypes of the Iranian BSF specimens to be identical to individuals from different parts of the world including Bhutan, China, Taiwan, Russia, Netherlands, France, Spain, UK, Kenya, Ghana, and USA, of Oriental, East Palearctic, Palearctic, Afrotropical, and Nearctic biogeographic regions respectively, when either barcode or 3’ end region of the COI gene (about 1500 bp totally) matched with the available data in GenBank or literature. Based on barcode region, the Iranian specimens correspond to the haplotype number one (I) as categorized by Ståhls et al. [21] and the haplotype C as categorized by Guilliet et al [44]. The later showed that haplotype C is a commercial haplotype and includes all the sequences originated from farmed strains of the industry working on BSF. The Iranian BSF haplotype matches with what is most commonly found in a farming context worldwide, however, there is no BSF commercial breeding site in the vicinity of the spot where the investigated individuals were captured in the wild. Finally, based on the phylogenetic analysis of this study and findings of Kaya et al. [2] we can conclude the Iranian BSF population is originated from North American strains.

Further efforts should investigate the overall presence of this fly in the country, particularly in southern Iran, close to the coasts of the Persian Gulf and Oman Sea, which have many active international trading ports, and to which the fly may already have been introduced by ships from different parts of the world.

Conclusion

We have provided documents on the presence of *H. illucens* from North of Iran. The high genetic similarity of the Iranian BSF with commercial strains from different bio-geographical regions suggests that the extensive human-mediated translocations of the BSF have led to genetically uniform domesticated strains in various parts of the World that originated from North America. These data are important for managing and producing commercial BSF colonies to support and

maintain the industries in the country that use BSF as a service provider e.g. feed, AMPs, fertilizer, and waste management processes.

Acknowledgment:

We are grateful to Dr. Martin Hauser, Senior Insect Biosystematist, from California Department of Food and Agriculture, Plant Pest Diagnostics Branch, USA for approving the species identification. We thank Mohammadreza Havasi, Fahime Rastegar, Talie Torabi Moheb and Amir Nikrooz for helping us in the field and laboratory. The manuscript was edited by the ICGEB Editing service (manuscripts@icgeb.org).

Funding: This research was supported by Tehran University of Medical Sciences, Iran.

Conflict of interest: We declare that we have no conflict of interest.

Compliance with Ethical Standards: This article does not contain any studies with human participants or animals performed by any of the authors. The protocols were conducted in this study followed the guidelines of the institutional ethical committee (University of Tehran).

References

1. Hoffmann L, Hull KL, Bierman A, Badenhorst R, Bester-van der Merwe AE, Rhode C (2021) Patterns of Genetic Diversity and Mating Systems in a Mass-Reared Black Soldier Fly Colony. Insects 12(6):480. https://doi.org/10.3390/insects12060480.
2. Kaya C, Generalovic TN, Ståhls G, Hauser M, Samayoa AC, Nunes-Silva CG, Roxburgh H, Wohlfahrt J, Ewusie EA, Kenis M, Hanboonsong Y (2021) Global population genetic structure and demographic trajectories of the black soldier fly, *Hermetia illucens*. BMC Biol 19(1):1-22. https://doi.org/10.1186/s12915-021-01029-w
3. Barragan-Fonseca KB, Dicke M, van Loon JJ (2017) Nutritional value of the black soldier fly (*Hermetia illucens* L.) and its suitability as animal feed–a review. J Insects Food Feed 3(2):105-120. https://doi.org/10.3920/JIFF2016.0055
4. Borel P, Hammaz F, Morand-Laffargue L, Creton B, Halimi C, Sabatier D, Desmarchelier C (2021) Using black soldier fly larvae reared on fruits and vegetables waste as a sustainable dietary source of provitamin a carotenoid. Food Chem X 359:129911. https://doi.org/10.1016/j.foodchem.2021.129911

5. Heuel M, Sandrock C, Leiber F, Mathys A, Gold M, Zurbrügg C, Gangnat IDM, Kreuzer M, Terranova M (2021) Black soldier fly larvae meal and fat can completely replace soybean cake and oil in diets for laying hens. Poult Sci. 100(4):101034. https://doi.org/10.1016/j.psj.2021.101034
6. Surendra KC, Tomberlin JK, van Huis A, Cammack JA, Heckmann LHL, Khanal, SK (2020) Rethinking organic wastes bioconversion: Evaluating the potential of the black soldier fly (Hermetia illucens (L.)) (Diptera: Stratiomyidae)(BSF). Waste Manag 117:58-80. https://doi.org/10.1016/j.wasman.2020.07.050
7. Gligorescu A, Fischer CH, Larsen PF, Nørgaard JV, Heckman L-HL (2020) Production and Optimization of Hermetia illucens (L.) Larvae Reared on Food Waste and Utilized as Feed Ingredient. Sustainability. 12(23):9864. https://doi.org/10.3390/su12239864
8. FAO - Food and Agriculture Organization of the United Nations (2013) Edible insects. Future prospects for food and feed security. In: Huis A. van, Itterbeeck J van, Klunder H, Mertens E, Halloran A, Muir G, Vantomme P, eds. Available from: http://www.fao.org/docrep/018/i3253e/i3253e00.htm.
9. Belghit I, Liland NS, Gjesdal P, Biancarosa I, Menchetti E, Li Y, Waagbø R, Krogdahl Å, Lock EJ (2019) Black soldier fly larvae meal can replace fish meal in diets of sea-water phase Atlantic salmon (Salmo salar). Aquaculture 503: 609-619. https://doi.org/10.1016/j.aquaculture.2018.12.032
10. Bellezza Oddon S, Biasato I, Imarisio A, Pipan M, Dekleva D, Colombino E, Capucchio MT, Meneguz M, Bergagna S, Barbero R, Gariglio M, Dabbou S, Fiorilla E, Gasco L, Schiavone A (2021) Black soldier fly and yellow mealworm live larvae for broiler chickens: Effects on bird performance and health status. J Anim Physiol Anim Nutr (Berl). 105 Suppl 1(Suppl 1):10-18. https://doi.org/10.1111/jpn.13567
11. Lord WD, Goff ML, Adkins TR, Haskell NH (1994) The black soldier fly *Hermetia illucens* (Diptera: Stratiomyidae) as a potential measure of human postmortem interval: observations and case histories. J Forensic 39(1):215-222.
12. Sheppard DC, Tomberlin JK, Joyce JA, Kiser BC, Sumner SM (2002) Rearing methods for the black soldier fly (Diptera: Stratiomyidae). J Med Entomol 39(4):695-698. https://doi.org/10.1603/0022-2585-39.4.695
13. Popa R, Green TR (2012) Using black soldier fly larvae for processing organic leachates. J Econ Entomol 105(2), pp.374-378. https://doi.org/10.1603/EC11192
14. Nguyen TT, Tomberlin JK, Vanlaerhoven S (2015) Ability of black soldier fly (Diptera: Stratiomyidae) larvae to recycle food waste. Environ Entomol 44(2):406-410. https://doi.org/10.1093/ee/nvv002
15. Mohamed H, Marusich E, Afanasev Y, Leonov S (2021) Fatty Acids-Enriched Fractions of *Hermetia illucens* (Black Soldier Fly) Larvae Fat Can Combat MDR Pathogenic Fish Bacteria Aeromonas spp. Int J Mol Sci 22(16):8829. https://doi.org/10.3390/ijms22168829
16. Park SI, Kim JW, Yoe SM (2015) Purification and characterization of a novel antibacterial peptide from black soldier fly (Hermetia illucens) larvae. Dev Comp Immunol 52(1):98-106. https://doi.org/10.1016/j.dci.2015.04.018
17. Xia J, Ge C, Yao H (2021) Antimicrobial Peptides from Black Soldier Fly (*Hermetia illucens*) as Potential Antimicrobial Factors Representing an Alternative to Antibiotics in Livestock Farming. Animals 11(7):1937. https://doi.org/10.3390/ani11071937
18. Wang Y, Liao MQ, Wang YH, Gong Q, Xu W, Wang M, Zhang YN, Wang JF (2021) Application of Sarcosaprophagous Insects to Estimate the Postmortem Interval in 11 Cases. Fa Yi Xue Za Zhi. 37(3):332-337. https://doi.org/10.12116/j.issn.1004-5619.2021.410201

19. Adjavon FJ, Li X, Hu B, Dong L, Zeng H, Li C, Hu W (2021) Adult House Fly (Diptera: Muscidae) Response to Black Soldier Fly (Diptera: Stratiomyidae) Associated Substrates and Potential Volatile Organic Compounds Identification. Environ Entomol 50(5):1037-1044. https://doi.org/10.1093/ee/nvab073
20. Sheppard DC, Newton GL, Thompson SA, Savage S (1994) A value-added manure management system using the black soldier fly. Bioresour Technol 50(3):275-279. https://doi.org/10.1016/0960-8524(94)90102-3
21. Ståhls G, Meier R, Sandrock C, Hauser M, Šašić Zorić L, Laiho E, Aracil A, Doderović J, Badenhorst R, Unadirekkul P, Mohd Adom NAB, Wein L, Richards C, Tomberlin JK, Rojo S, Veselić S, Parviainen T (2020) The puzzling mitochondrial phylogeography of the black soldier fly (*Hermetia illucens*), the commercially most important insect protein species. BMC Ecol Evol 20:1-10. https://doi.org/10.1186/s12862-020-01627-2
22. Marshall SA, Woodley NE, Hauser M (2015) The historical spread of the black soldier fly, *Hermetia illucens* (L.) (Diptera, Stratiomyidae, Hermetiinae), and its establishment in Canada. J Entomol Soc Ont 146:51–4.
23. Leclercq M (1997) About *Hermetia illucens* (Linnaeus, 1758) (soldier fly) (Diptera, Stratiomyidae: Hermetiinae). Bulletin et Annales de la Societe Royale Belge d'Entomologie (Belgium) 133:275–82.
24. Simon C, Frati F, Beckenbach A, Crespi B, Liu H, Flook P (1994) Evolution, weighting, and phylogenetic utility of mitochondrial gene sequences and a compilation of conserved polymerase chain reaction primers. Annu Rev Entomol 87(6):651–701. https://doi.org/10.1093/aesa/87.6.651
25. Hebert PD, Ratnasingham S, De Waard JR (2003) Barcoding animal life: cytochrome c oxidase subunit 1 divergences among closely related species. Philos Trans R Soc Lond B Biol Sci 270(suppl_1): S96-S99. https://doi.org/10.1098/rsbl.2003.0025
26. Karimian F, Oshaghi MA, Sedaghat MM, Waterhouse RM, Vatandoost H, Hanafi-Bojd AA, Ravasan NM, Chavshin AR (2014) Phylogenetic analysis of the oriental-Palearctic-Afrotropical members of Anopheles (Culicidae: Diptera) based on nuclear rDNA and mitochondrial DNA characteristics. Jpn J Infect Dis 67(5): 361–367. https://doi.org/10.7883/yoken.67.361
27. Hashemi-Aghdam SS, Rafie G, Akbari S, Oshaghi MA (2017) Utility of mtDNA-COI Barcode Region for Phylogenetic Relationship and Diagnosis of Five Common Pest Cockroaches. J Arthropod Borne Dis 11(2):182-193.
28. Heydari A, Oshaghi MA, Nazari A, Shayeghi M, Sanatgar E (2020) Genetic structure of rice striped stem borer, *Chilo suppressalis* (Lepidoptera:Crambidae) in North of Iran. Mitochondrial DNA A DNA Mapp Seq Anal 31(8):327-334. https://doi.org/10.1080/24701394.2020.1815718
29. Oshaghi MA, Yaaghoobi F, Abaie MR (2006) Pattern of mitochondrial DNA variation between and within *Anopheles stephensi* (Diptera: Culicidae) biological forms suggests extensive gene flow. Acta Trop 99(2-3):226-33. https://doi.org/10.1016/j.actatropica.2006.08.005
30. Oshaghi MA, Shemshad Kh, Yaghobi-Ershadi MR, Pedram M, Vatandoost H, Abaie MR, Akbarzadeh K, Mohtarami F (2007) Genetic structure of the malaria vector *Anopheles superpictus* in Iran using mitochondrial cytochrome oxidase (COI and COII) and morphologic markers: a new species complex? Acta Trop 101(3):241-8. https://doi.org/10.1016/j.actatropica.2007.02.006
31. Bazrafkan S, Vatandoost H, Heydari A, Bakhshi H, Panahi-Moghadam S, Hashemi-Aghdam S, Mohtarami F, Rahimiforoushan A, Anlaandş S, Shayeghi M, Oshaghi MA, Abtahi SM (2016) Discrimination of *Paederus fuscipes* and *Paederus littoralis* by mtDNA-COI PCR-RFLP. J Arthropod Borne Dis 10(4):454-461.

32. Koosha M, Oshaghi MA, Sedaghat MM, Vatandoost H, Azari-Hamidian S, Abai MR, Hanafi-Bojd AA, Mohtarami F (2017) Sequence analysis of mtDNA COI barcode region revealed three haplotypes within *Culex pipiens* assemblage. Exp Parasitol 181:102-110. https://doi.org/10.1016/j.exppara.2017.08.003
33. Talebzadeh F, Oshaghi MA, Akbarzadeh K, Panahi-Moghadam S (2020) Molecular Species Identification of Six Forensically Important Iranian Flesh Flies (Diptera). J Arthropod Borne Dis 14(4):416-424. https://doi.org/10.18502/jad.v14i4.5279
34. Khanzadeh F, Khaghaninia S, Maleki-Ravasan N, Oshaghi MA, Adler PH (2020) Black flies (Diptera: Simuliidae) of the Aras River Basin: Species composition and floral visitation. Acta Trop 209:105536. https://doi.org/10.1016/j.actatropica.2020.105536
35. Hollingsworth, P M, Graham S W, Little, DP (2011) Choosing and Using a Plant DNA Barcode. PLoS ONE 6(5): e19254. https://doi.org/10.1371/journal.pone.0019254
36. Heydari A, Anlaş S, Bakhshi H, Koosha M, Choubdar N, Panahi-Moghadam S, Oshaghi MA (2023) Molecular Characterization of *Paederus* Spp (Coleoptera: Staphylinidae, Paederinae) the Agent of Human Linear Dermatitis in the Caspian Sea Coast, North of Iran. J Arthropod Borne Dis 17(1):94-104. https://doi.org/10.18502/jad.v17i1.13206
37. Hosseini-Chegeni A, Nasrabadi M, Sadat Hashemi-Aghdam S, Oshaghi MA, Lotfi A, Telmadarraiy Z, Sedaghat MM (2019) Molecular identification of *Rhipicephalus* species (Acari: Ixodidae) parasitizing livestock from Iran. Mitochondrial DNA A DNA Mapp Seq Anal 30(3):448-456. https://doi.org/10.1080/24701394.2018.1546298
38. Moin-Vaziri V, Oshaghi MA, Yaghoobi-Ershadi MR, Derakhshandeh-Peykar P, Abaei MR, Mohtarami F, Zahraei-Ramezani AR, Nadim A (2016) ITS2-rDNA Sequence Variation of *Phlebotomus sergenti* s.l. (Dip: Psychodidae) Populations in Iran. J Arthropod Borne Dis 10(4):462-473.
39. Oshaghi MA, Yaghobi-Ershadi MR, Shemshad K, Pedram M, Amani H (2008) The *Anopheles superpictus* complex: introduction of a new malaria vector complex in Iran. Bull Soc Pathol Exot 101(5):429-34.
40. Oshaghi MA, Vatandoost H, Gorouhi A, Abai MR, Madjidpour A, Arshi S, Sadeghi H, Nazari M, Mehravaran A (2011) Anopheline species composition in borderline of Iran-Azerbaijan. Acta Trop119(1):44-9. https://doi.org/10.1016/j.actatropica.2011.04.005
41. De Carvalho CJB, De Mello-Patiu CA (2008) Key to the adults of the most common forensic species of Diptera in South America. Revista Brasileira de Entomologia 52(3):390–406. https://doi.org/10.1590/S0085-56262008000300012
42. Folmer O, Black M, Hoeh W, Lutz R, Vrijenhoek R (1994) DNA primers for amplification of mitochondrial cytochrome c oxidase subunit I from diverse metazoan invertebrates. Mol Mar Biol Biotechnol 3:294–299.
43. Sievers F, Wilm A, Dineen D, Gibson TJ, Karplus K, Li W, ... & Higgins DG. (2011). Fast, scalable generation of high-quality protein multiple sequence alignments using Clustal Omega. Mol Sys Biol 7(1):539. https://doi.org/10.1038/msb.2011.75
44. Guilliet J, Baudouin G, Pollet N, Filée J (2022) What complete mitochondrial genomes tell us about the evolutionary history of the black soldier fly, *Hermetia illucens*. BMC Ecol Evol 22(1):72. https://doi.org/10.1186/s12862-022-02025-6
45. Kumar S, Stecher G, Li M, Knyaz C, Tamura K (2018) MEGA X: Molecular Evolutionary Genetics Analysis across Computing Platforms. Mol Biol Evol 35:1547-1549. https://doi.org/10.1093/molbev/msy096

46. Qi Y, Xu J, Tian X, Bai Y, Gu X (2017) The complete mitochondrial genome of *Hermetia illucens* (Diptera: Stratiomyidae). Mitochondrial DNA B Resour 2(1):189-190. https://doi.org/10.1080/23802359.2017.1307708
47. Soyeon P, Hansu Ch, Ji-young Ch, Gilsang J (2017) Population Structure of the Exotic Black Soldier Fly, *Hermetia illucens* (Diptera: Stratiomyidae) in Korea. Korean J Envirn Ecol 31(6):520-528. https://doi.org/10.13047/KJEE.2017.31.6.520
48. Khamis FM, Ombura FLO, Akutse KS, Subramanian S, Mohamed SA, Fiaboe KKM, Saijuntha W, Van Loon JJA, Dicke M, Dubois T, Ekesi S, Tanga CM (2020) Insights in the Global Genetics and Gut Microbiome of Black Soldier Fly, *Hermetia illucens*: Implications for Animal Feed Safety Control. Front Microbiol 11:1538. https://doi.org/10.3389/fmicb.2020.01538
49. Fatehian S, Jelokhani-Niaraki M, Kakroodi AA, Dero QY, Samany NN (2018) A volunteered geographic information system for managing environmental pollution of coastal zones: A case study in Nowshahr, Iran. Ocean Coast Manag 163:54-65. https://doi.org/10.1016/j.ocecoaman.2018.06.008
50. Sheppard C (1983) House fly and lesser fly control utilizing the black soldier fly in manure management systems for caged laying hens. Environ Entomol 12:1439–1442. https://doi.org/10.1093/ee/12.5.1439
51. Diener S, Solano NMS, Gutiérrez FR, Zurbrügg C, Tockner K (2011) Biological treatment of municipal organic waste using black soldier fly larvae. Waste Biomass Valorization 2(4):357-363. https://doi.org/10.1007/s12649-011-9079-1
52. Ewusie A, Kwapong EK, Ofosu-Budu P, Sandrock G,, Akumah C, Nartey A, Teye-Gaga E, Agyarkwah CK, Adamtey N (2018) Development of Black Soldier Fly, *Hermetia illucens* (Diptera: Stratiomyidae) in Selected Organic Market Waste Fractions in Accra, Ghana. Asian J Biotechnol Bioresour Technol 4(1):1–16. doi: 10.9734/AJB2T/2018/42371.
53. Myers HM, Tomberlin JK, Lambert BD, Kattes D (2014) Development of black soldier fly (Diptera: Stratiomyidae) larvae fed dairy manure. Environ Entomol 37(1):11-15. https://doi.org/10.1093/ee/37.1.11
54. Miranda CD, Cammack JA, Tomberlin JK (2019) Life-history traits of the black soldier fly, *Hermetia illucens* (L.) (Diptera: Stratiomyidae), reared on three manure types. Animals 9(5):281. https://doi.org/10.3390/ani9050281
55. Tomberlin JK, Sheppard DC, Joyce JA (2005) Black soldier fly (Diptera: Stratiomyidae) colonization of pig carrion in South Georgia. J Forensic Sci 50(1):JFS2003391-2.
56. Harnden LM, Tomberlin JK (2016) Effects of temperature and diet on black soldier fly, *Hermetia illucens* (L.) (Diptera: Stratiomyidae), development. Forensic Sci Int Anim Environ 266:109-116. https://doi.org/10.1016/j.forsciint.2016.05.007
57. Liu X, Chen X, Wang H, Yang Q, ur Rehman K, Li W, Cai M, Li Q, Mazza L, Zhang J, Yu Z (2017) Dynamic changes of nutrient composition throughout the entire life cycle of black soldier fly. PLoS One 12(8): e0182601. https://doi.org/10.1371/journal.pone.0182601
58. Tahamtani FM, Ivarsson E, Wiklicky V, Lalander C, Wall H, Rodenburg TB, Tuyttens FA, Hernandez CE (2021) Feeding live Black Soldier Fly larvae (*Hermetia illucens*) to laying hens: effects on feed consumption, hen health, hen behavior, and egg quality. Poult Sci 100(10):101400. https://doi.org/10.1016/j.psj.2021.101400
59. Pazmiño MíF, Del Hierro AG, Flores FJ (2023) Genetic diversity and organic waste degrading capacity of *Hermetia illucens* from the evergreen forest of the Equatorial Choco lowland. PeerJ 11:e14798 http://doi.org/10.7717/peerj.14798

60. Maquart PO, Richard D, Willems J, (2020) First record of the Black Soldier Fly, *Hermetia illucens*, in the Western regions of France (Vendée, Loire-Atlantique, Ille-et-Vilaine) with notes on its worldwide repartition (Diptera, Stratiomyidae). Bulletin de la Société entomologique de France 125(1):13-18. https://doi.org/10.32475/bsef_2104
61. Linton YM, Samanidou-Voyadjoglou A, Harbach RE (2002) Ribosomal ITS2 sequence data for *Anopheles maculipennis* and *An. messeae* in northern Greece, with a critical assessment of previously published sequences. Insect Mol Biol 11:379–383.
62. Sandrock C, Leupi S, Wohlfahrt J, Kaya C, Heuel M, Terranova M, Blanckenhorn WU, Windisch W, Kreuzer M, Leiber F (2022) Genotype-by-Diet Interactions for Larval Performance and Body Composition Traits in the Black Soldier Fly, *Hermetia illucens*. Insects 13(5):424. https://doi.org/10.3390/insects13050424
63. Malekian M, And Homami, R (2012) Fundamentals of Conservation Biology, Mashhad University Jihad Publications. Mashhad, 304 pages.
64. Cochet A, Calba C, Jourdain F, Grard G, Durand GA, Guinard A, Investigation Team; Noël H, Paty MC, Franke F (2022) Autochthonous dengue in mainland France, 2022: Geographical extension and incidence increase. Euro Surveill 27:2200818. https://doi.org/10.2807/1560-7917.ES.2022.27.44.2200818
65. Wilcove D, Rothstein D, Dublow J, Phillips A, Losos E (1998) Quantifying threats to imperiled species in the United States. Biosci 48:607–615.

Statements and Declarations

The authors have no relevant financial or non-financial interests to disclose.

Authors contribution: All authors contributed to the study conception and design. Material preparation, data collection and analysis were performed by Afrooz Boukanand, Katayoun Pahlavan Yali, Fahimeh Talebzadeh, and Nazanin Naseri Karimi. The first draft of the manuscript was written by Afrooz Boukan and all authors commented on previous versions of the manuscript. All authors read and approved the final manuscript.

Data Availability statement

All data generated or analysed during this study are included in this published article.

Figures and their legends

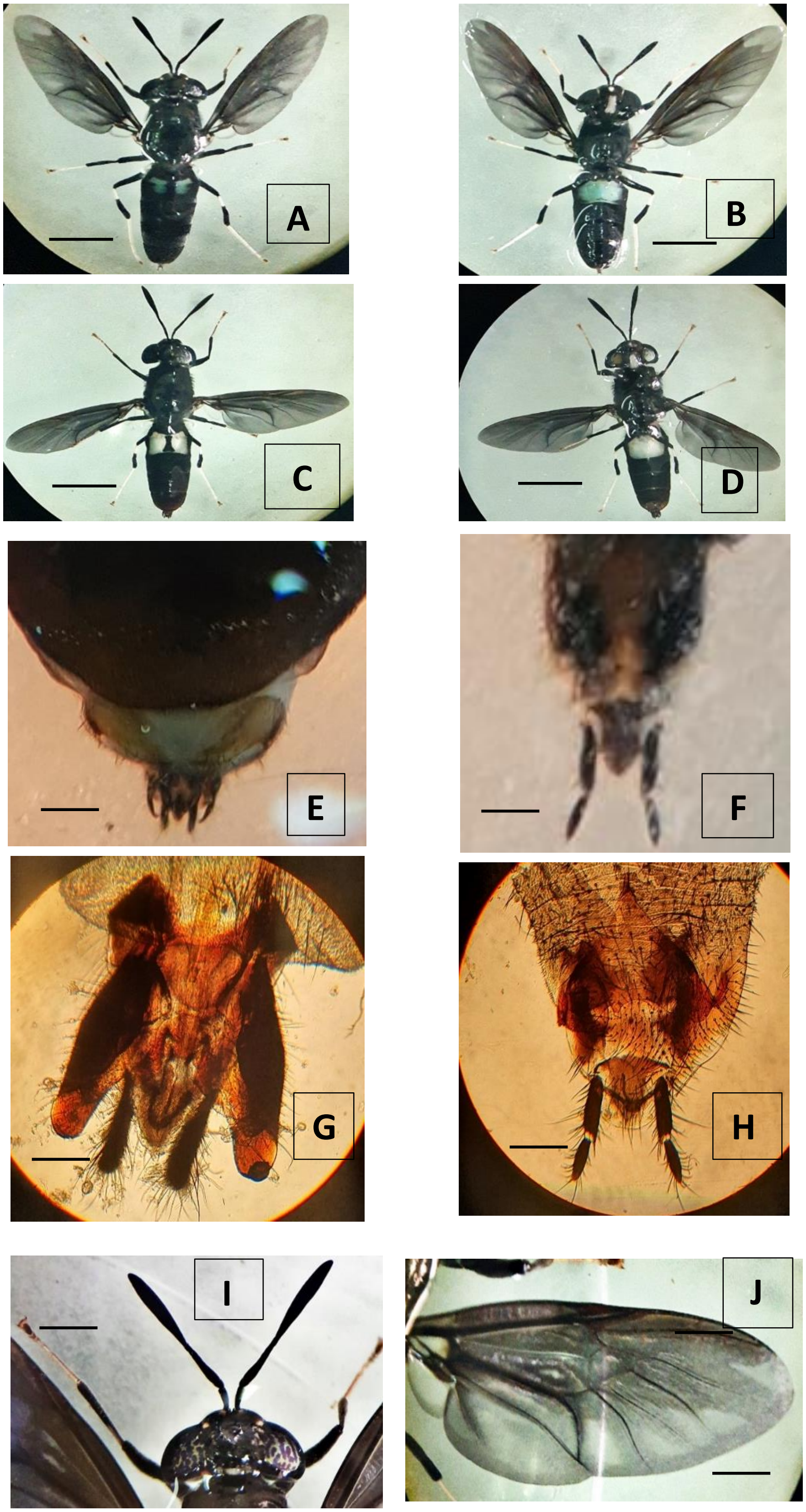
A
B
C
D
E
F
G
H
I
J

Figure 1. Morphological characteristics of *Hermetia illucens* captured in northern Iran, 2020. A, Dorsal view of female. B, Ventral view of female. C, Dorsal view of male. D. Ventral view of male. E & G, Tip of abdomen and genital capsule (10x), male. Scale represents 2mm. F & H, Tip of abdomen and Genital structure (10x), female, dorsal view. I, Dorsal view of the head. J, Wing.

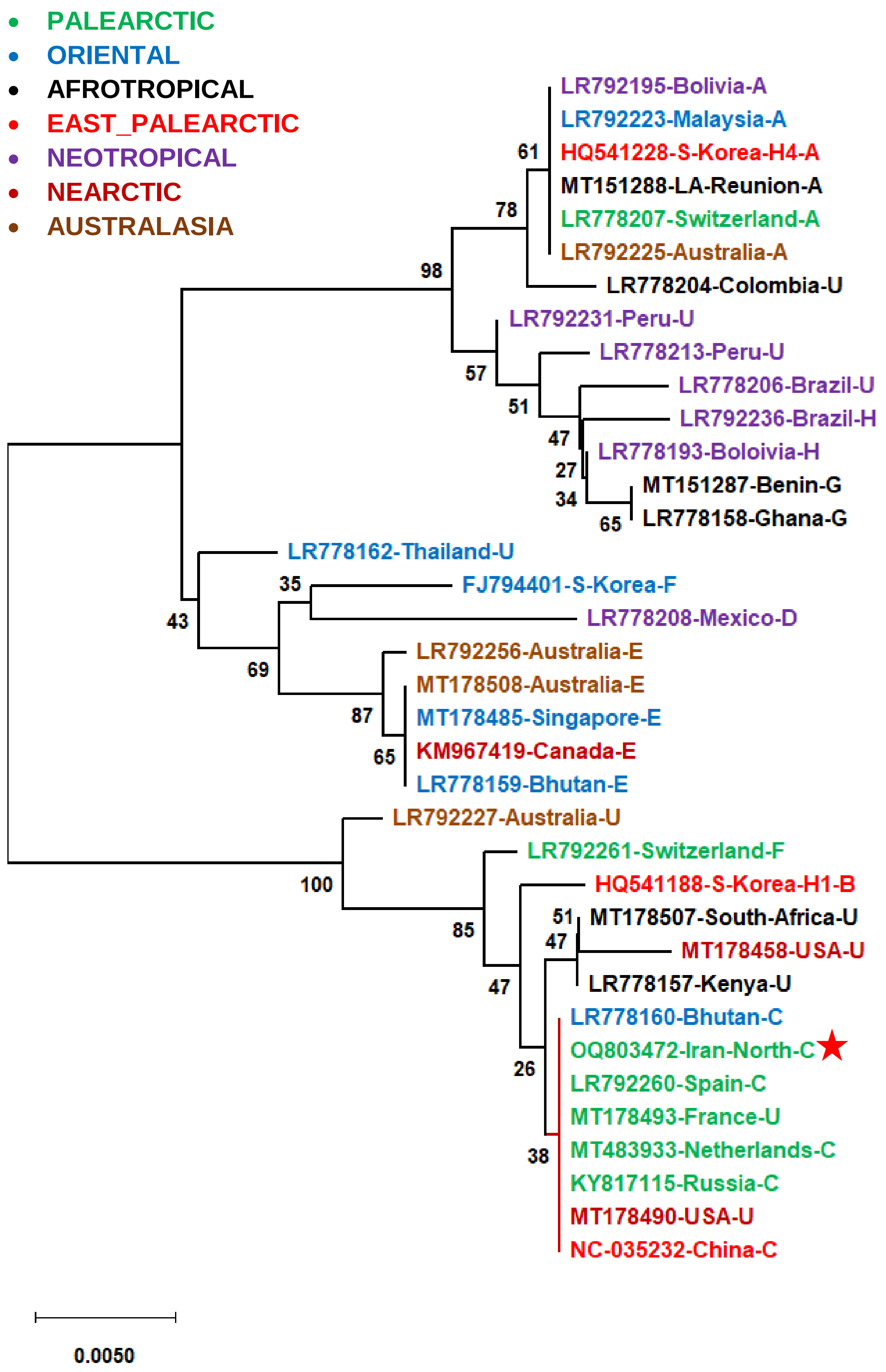
PALEARCTIC
ORIENTAL
AFROTROPICAL
EAST_PALEARCTIC
NEOTROPICAL
NEARCTIC
AUSTRALASIA
LR792195-Bolivia-A
LR792223-Malaysia-A
HQ541228-S-Korea-H4-A
MT151288-LA-Reunion-A
LR778207-Switzerland-A
LR792225-Australia-A
LR778204-Colombia-U
LR792231-Peru-U
LR778213-Peru-U
LR778206-Brazil-U
LR792236-Brazil-H
LR778193-Boloivia-H
MT151287-Benin-G
LR778158-Ghana-G
LR778162-Thailand-U
FJ794401-S-Korea-F
LR778208-Mexico-D
LR792256-Australia-E
MT178508-Australia-E
MT178485-Singapore-E
KM967419-Canada-E
LR778159-Bhutan-E
LR792227-Australia-U
LR792261-Switzerland-F
HQ541188-S-Korea-H1-B
MT178507-South-Africa-U
MT178458-USA-U
LR778157-Kenya-U
LR778160-Bhutan-C
OQ803472-Iran-North-C
LR792260-Spain-C
MT178493-France-U
MT483933-Netherlands-C
KY817115-Russia-C
MT178490-USA-U
NC-035232-China-C
61
78
98
57
51
47
27
34
65
35
43
69
87
65
100
85
51
47
47
26
38
0.0050

Figure 2 The phylogenetic tree inferred by using Neighbor-Joining method based on 628 bp of the barcode regions of COI gene of *Hermetia illucens*. The Iranian representative is labeled with red star. The other data are representatives of available sequences in GenBank and are labelled with country and haplotype (A-H, U represents unknown) [44]. The bootstrap values of more than 50 are shown on the internal nodes. The colors of the branches correspond to the biogeographical regions.

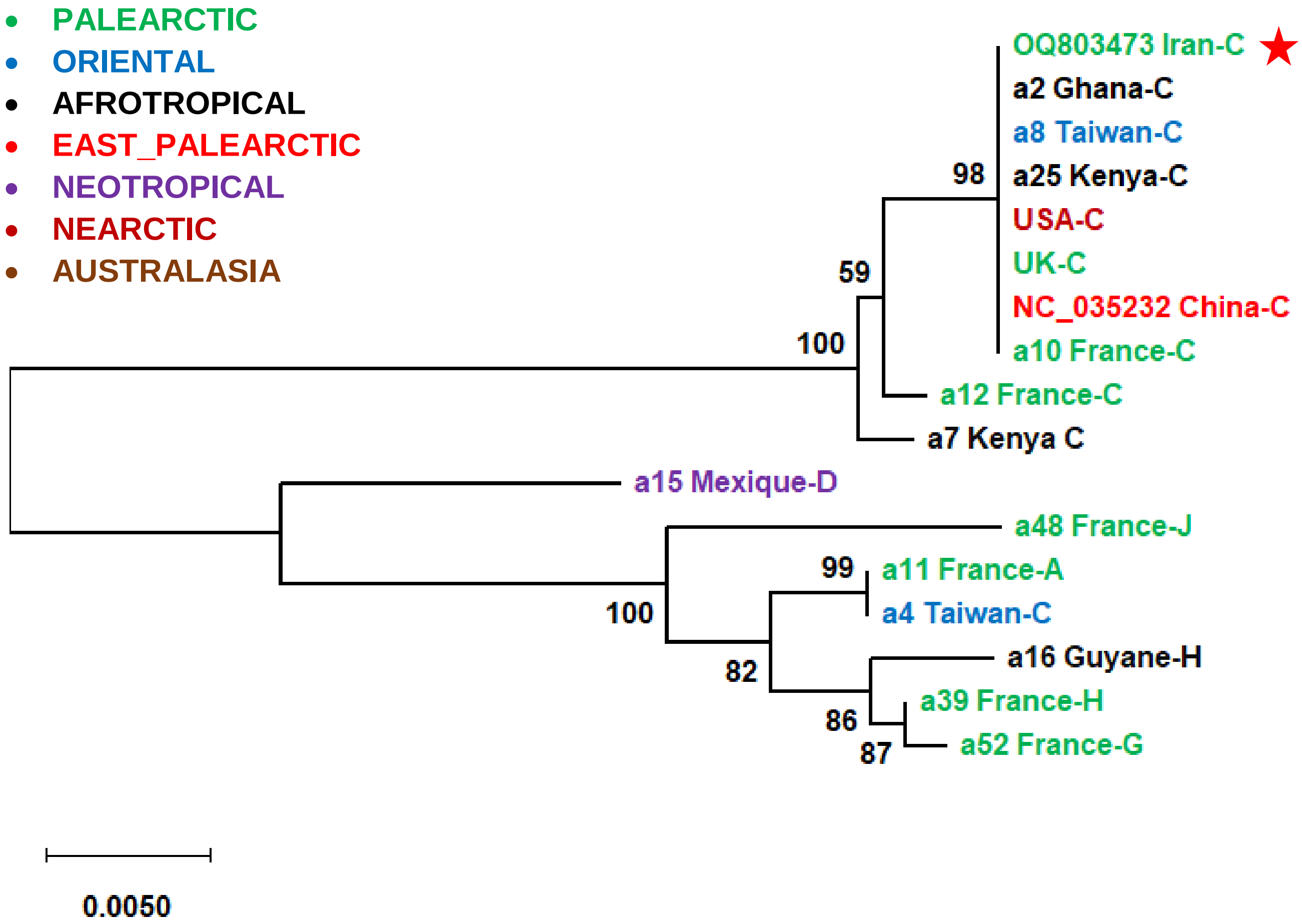

Figure 3 The phylogenetic tree inferred by using Neighbor-Joining method based on 840 bp of the 3'-end of COI gene of *Hermetia illucens*. The Iranian BSF is labeled with red star. The other

data are representatives of available sequences in GenBank and Guilliet et al [44] and are labelled with code number, country, and haplotype. The colors of the branches correspond to the biogeographical regions. This tree was made by keeping only one or two sequences per haplotype except for Haplotype C. The bootstrap values are shown on the internal nodes.
